# Altered anterior cingulate cortex subregional connectivity associated with cognitions for distinguishing the spectrum of pre-clinical Alzheimer’s disease

**DOI:** 10.3389/fnagi.2022.1035746

**Published:** 2022-12-07

**Authors:** Qianqian Yuan, Xuhong Liang, Chen Xue, Wenzhang Qi, Shanshan Chen, Yu Song, Huimin Wu, Xulian Zhang, Chaoyong Xiao, Jiu Chen

**Affiliations:** ^1^Department of Radiology, The Affiliated Brain Hospital of Nanjing Medical University, Nanjing, China; ^2^Department of Radiology, Drum Tower Hospital, Medical School of Nanjing University, Nanjing, China; ^3^Institute of Medical Imaging and Artificial Intelligence, Nanjing University, Nanjing, China; ^4^Medical Imaging Center, The Affiliated Drum Tower Hospital, Medical School of Nanjing University, Nanjing, China

**Keywords:** Alzheimer’s disease, anterior cingulate cortex, functional connectivity, subjective cognitive decline, mild cognitive impairment

## Abstract

**Background:**

Subjective cognitive decline (SCD) and amnestic mild cognitive impairment (aMCI) are considered part of the early progression continuum of Alzheimer’s disease (AD). The anterior cingulate cortex (ACC), a hub of information processing and regulation in the brain, plays an essential role in AD pathophysiology. In the present study, we aimed to systematically identify changes in the functional connectivity (FC) of ACC subregions in patients with SCD and aMCI and evaluate the association of these changes with cognition.

**Materials and methods:**

Functional connectivity (FC) analysis of ACC sub-regions was performed among 66 patients with SCD, 71 patients with aMCI, and 78 healthy controls (HCs). Correlation analyses were performed to examine the relationship between FC of altered ACC subnetworks and cognition.

**Results:**

Compared to HCs, SCD patients showed increased FC of the bilateral precuneus (PCUN) and caudal ACC, left superior frontal gyrus (SFG) and subgenual ACC, left inferior parietal lobule (IPL) and dorsal ACC, left middle occipital gyrus (MOG) and dorsal ACC, and left middle temporal gyrus (MTG) and subgenual ACC, while aMCI patients showed increased FC of the left inferior frontal gyrus (IFG) and dorsal ACC and left medial frontal gyrus (MFG) and subgenual ACC. Compared to patients with SCD, patients with aMCI showed increased FC of the right MFG and dorsal ACC and left ACC and subgenual ACC, while the left posterior cingulate cortex (PCC) showed decreased FC with the caudal ACC. Moreover, some FC values among the altered ACC subnetworks were significantly correlated with episodic memory and executive function.

**Conclusion:**

SCD and aMCI, part of the spectrum of pre-clinical AD, share some convergent and divergent altered intrinsic connectivity of ACC subregions. These results may serve as neuroimaging biomarkers of the preclinical phase of AD and provide new insights into the design of preclinical interventions.

## Introduction

Subjective cognitive decline (SCD) refers to cognitive impairment in elderly patients who report a decline in subjective memory compared with previous levels (Xue et al., 2019). Mild cognitive impairment (MCI), particularly amnestic MCI (aMCI), is a transitional state between normal cognition and Alzheimer’s disease (AD) (Chen et al., 2020). Both conditions are considered part of the preclinical continuum of AD (Wang et al., 2020, 2021). Many studies have shown that individuals progress through stages from SCD to aMCI, finally to AD (Berger-Sieczkowski et al., 2019). Notably, the different stages of AD may be associated with different pathological changes. Neuroimaging can help reveal the pathological mechanisms of AD progression (Wang et al., 2021).

Functional magnetic resonance imaging (fMRI) can be used to assess large-scale neural systems that exhibit spontaneous synchronized fluctuations (Peng et al., 2021; Yuan et al., 2021). Functional correlation of fMRI signals between brain regions acquired during the resting state is considered a reliable method for understanding age-related changes in cognitive function and emotional processing (Xu et al., 2021; Xue et al., 2021; Liang et al., 2022). In recent years, resting-state functional connectivity (rsFC) has been widely used to study changes in brain function due to aging and in preclinical stages related to dementia. The cingulate cortex, which is the thick cortical band surrounding the corpus callosum, is considered one of the most prominent features on the mesial surface of the brain (Shackman et al., 2011). Many studies on depression have examined the anterior cingulate cortex (ACC) and shown it to be involved in information processing and regulation (Kelly et al., 2009; Shackman et al., 2011; Peng et al., 2021). And the degenerative changes in ACC are consistently seen in Alzheimer’s disease (AD) (Jeong et al., 2021). At the same time, some studies have shown that the change of ACC subregional thickness is an effective predictor of SCD and aMCI conversion to AD (Sambuchi et al., 2015; Jung et al., 2019; Jeong et al., 2021). Although the data are more limited, there are also studies supporting involvement of the ACC in top-down functions and cognition in internalized conditions (Sheena et al., 2021). Thus, it is reasonable to think that the ACC is associated with functional changes in preclinical AD.

Previous rsFC studies have shown that the ACC can be subdivided into caudal, dorsal, rostral, peripheral, and subregional regions. These ACC subregions are believed to play key roles in cognitive control, cognitive and executive goal orientation, and emotional and affective processes (Shin and Liberzon, 2010; Cera et al., 2019). The ACC is considered a component of the dorsal attention network (DAN), which is believed to be the foundation of neural aging (Cera et al., 2019); however, the hippocampus and ACC are also components of the limbic system. The hippocampus is a key structure in episodic memory (Rolls, 2018), while the ACC subregion is considered to play a key role in information regulation. Although data are limited, the fact remains that the anterior cingulate cortex (ACC) is involved in multiple cognitive processes, including executive function (Jung et al., 2019). At the same time, the loss of ACC volume has been confirmed in patients with aMCI converted to AD (Jeong et al., 2021); On fMRI, ACC also showed degenerative changes and reduced functional activity in patients with early AD (Jung et al., 2019). Therefore, studying the role of the ACC in the development of AD is of great significance (Behrens et al., 2009; Cera et al., 2019).

In the present study, we used neuroimaging to examine FC changes in patients with SCD and aMCI to enhance understanding of the pathogenesis of different stages of the disease and find effective methods for preclinical diagnosis and treatment of AD. To do so, we examined changes in FC patterns in the ACC among preclinical AD patients and further investigated the relationship patterns of FC changes and cognitive function. We hypothesized there are altered FC patterns of the ACC in SCD and aMCI patients, and that such patterns may be associated with different degrees of cognitive impairment in the preclinical stage of AD.

## Materials and methods

### Participants

A total of 223 elderly individuals participated in our study, including 81 healthy controls (HCs), 67 with SCD, and 75 with aMCI. Eight patients were excluded due to excessive head motion (cumulative translation or rotation > 3.0 mm or 3.0°). As a result, data from 215 participants (78 HC, 66 SCD, and 71 aMCI) were analyzed. Data were acquired from our in-house database, the Nanjing Brain Hospital Alzheimer’s Disease Spectrum Neuroimaging Project (NBH-ADsnp-2) (Nanjing, China), which is continuously updated.

The inclusion criteria for the HC group were as follows: (a) no memory impairment; (b) normal cognitive level corresponding to age and years of education; (c) clinical dementia score (CDR) = 0; (d) Mini-Mental State Examination (MMSE) score ≥ 26; and (e) aged 50–80 years (Chen et al., 2016b; Gu et al., 2018; Yan et al., 2018). The inclusion criteria for the SCD group were based on the published SCD criteria proposed by the subjective cognitive decline initiative (SCD-I) (Jessen et al., 2014): (a) self-reported persistent memory decline; (b) a Subjective Cognitive Decline Questionnaire (SCD-Q) score > 5; (c) MMSE and Montreal Cognitive Assessment (MOCA) scores within the normal range (appropriate for age and education level); (d) CDR = 0; (e) aged 50–80 years; (f) and a Hamilton Depression Scale (HAMD) score < 7 (Dillen et al., 2017; Cedres et al., 2019). Lastly, the inclusion criteria for the aMCI group were: (a) a chief complaint of memory impairment for 3 months or more confirmed by patients or their relatives; (b) objective memory impairment (consistent with age and education level); (c) normal general cognitive function, MMSE score ≥ 24; (d) no or minimal impairment in activities of daily living; (e) CDR = 0.5; (f) aged 50–80 years; (g) no dementia; and (h) a HAMD score ≥ 7. These inclusion criteria include diagnostic criteria defined in previous studies and revised consensus criteria in subsequent reports (Petersen et al., 1999; Winblad et al., 2004; Huang et al., 2019).

Unqualified subjects were excluded by the following exclusion criteria: (a) history of stroke, head injury, alcoholism, brain tumor, Parkinson’s disease, encephalitis, major depressive disorder (HAMD was excluded), or other neurological or psychiatric disorders (clinical evaluation and medical history were excluded); (b) a diagnosis of a major medical condition (such as cancer, thyroid dysfunction, anemia, syphilis, etc.); (c) severe loss of sight or hearing; (d) inability to complete neuropsychological assessment or contraindications to MRI; and (e) T2-weighted MRI showing major changes in white matter (WM), infarction, or other lesions (Chen et al., 2019). The above inclusion and exclusion criteria are similar to those used in our previous studies (Chen et al., 2016b,^2019^, ^2020^; Xue et al., 2021; Song et al., 2022).

This study was approved by the head of the Brain Hospital affiliated to Nanjing Medical University and the ethics committee. All participants provided written informed consent.

### Neurocognitive assessments

All participants underwent a comprehensive and standardized assessment that included the MMSE, ADL, MoCA, MDRS-2, SCD-Q, CDR, HAMD, Hachinski Ischemic Scale (HIS), Auditory Verbal Learning Test (AVLT; including the AVLT-immediate, AVLT-5 min delay, and AVLT-20 min delay), CFT, Logical Memory Test (LMT), Rey Complex Figure Test (CFT) delay, Clock-Drawing Test (CDT), Category Verbal Fluency Test (including CVFT-animals and CVFT-objects), Boston Naming Test, Symbol Digit Modalities Test, part A and B of the Trail Making Test (TMT), part A, B, and C of the Stroop Test, Digit Span Test (including DS forward and DS backward), and the Semantic Similarity Test. The assessment was validated by two senior neuropsychologists and carried out by two experienced clinicians. General cognitive function, episodic memory, information processing speed, executive function, and visuospatial function were also evaluated (Gu et al., 2017; Gao et al., 2018). This neuropsychological assessment method is similar to that used in our previous studies (Chen et al., 2015, 2016a; Xue et al., 2019).

### Magnetic resonance imaging data acquisition

Details about the data acquisition process and image acquisition parameters of NBH-ADsnp-2 are described in our previous studies (Chen et al., 2016a,^2019^, ^2020^; Xue et al., 2019). Briefly, MRI data were acquired using a 3.0 Tesla Verio Siemens scanner with an 8-channel head-coil at the Affiliated Brain Hospital of Nanjing Medical University (Nanjing, China). Participants were instructed to rest with their eyes open, to not fall asleep, and to not think of anything in particular during the resting-state scans. The gradient-echo echo-planar imaging (GRE-EPI) sequence included 240 volumes. The parameters were as follows: repetition time (TR) = 2,000 ms, echo time (TE) = 30 ms, number of slices = 36, thickness = 4.0 mm, gap = 0 mm, matrix = 64 × 64, flip angle (FA) = 90°, field of view (FOV) = 220 mm × 220 mm, acquisition bandwidth = 100 kHz, voxel size = 3.4 × 3.4 × 4 mm^3^. Imaging took approximately 8 min. High-resolution T1-weighted images were acquired by a 3D magnetization-prepared rapid gradient-echo (MPRAGE) sequence. The parameters were as follows: TR = 1,900 ms, TE = 2.48 ms, inversion time (TI) = 900 ms, number of slices = 176, thickness = 1.0 mm, gap = 0.5 mm, matrix = 256 × 256, FA = 9°, FOV = 256 mm × 256 mm, voxel size = 1 × 1 × 1 mm^3^. The imaging process took approximately 4.26 min.

### Image preprocessing

All fMRI data were preprocessed using MATLAB 2013b^1^ and Data Processing and Analysis for Brain Imaging (DPABI) (Yan et al., 2013, 2016), which is based on Statistical Parametric Mapping (SPM8)^2^. As the first step, the first 10 time points were removed to reduce instability of the MRI signal. Corrections were performed for intra-volume acquisition time differences among slices and inter-volume motion effects during the scan. Participants with excessive head motion (cumulative translation or rotation > 3.0 mm or 3.0°) were excluded. Next, we applied affine regularization in European segmentation and nuisance covariate regression with 24 motion parameters, a global signal, a white matter signal, and a cerebrospinal fluid signal (Ashburner and Friston, 2009; Fox et al., 2009). After nuisance covariate regression, functional images were normalized by DARTEL into Montreal Neurological Institute (MNI) space (resampling voxel size, 3 × 3 × 3 mm^3^) and spatially smoothed using a Gaussian kernel (Cha et al., 2013) of 6 mm^3^ full-width at half maximum (FWHM) to reduce spatial noise and differences in anatomical structures among subjects. Temporal band-pass filtering (0.01–0.1 Hz) was applied to reduce the effect of low-frequency drift and high-frequency physiological noise.

### Functional connectivity analysis

**FIGURE 1:**
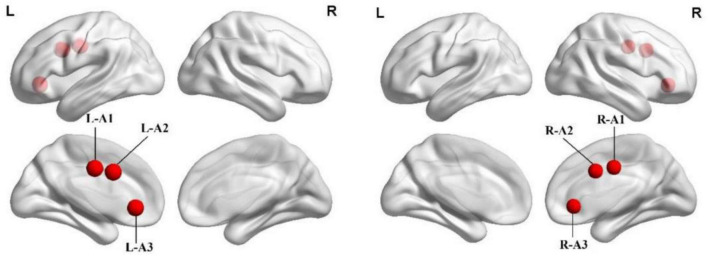
Spatial location of the anterior cingulate cortex subregions. A1, caudal anterior cingulate cortex; A2, dorsal anterior cingulate cortex; A3, subgenual anterior cingulate cortex; L, left; R, right.

In line with previous studies on ACC subdivisions (Kelly et al., 2009), three seeds were defined for each hemisphere in MNI space: caudal ACC (A1; MNI =± 5, −10, 37), dorsal ACC (A2; MNI =± 5, 10, 33), and subgenual ACC (A3; MNI =± 5, 34, −4) (Figure 1 and Table 1). We extracted the mean time series of all voxels in the seeds of each subject’s ACC subregion and used it as the reference time process. Next, we performed cross-correlation analysis of the voxel direction between each seed region and the whole brain within the gray matter (GM) mask. Finally, Fisher’s r-to-z transformation was performed to increase the normality of the correlation coefficients.

**TABLE 1:** Seeds and montreal neurological institute (MNI) coordinates.

Seeds	ACC sub-regions	MNI coordinates		
		
		X	Y	Z
A1	Caudal ACC	± 5	−10	37
A2	Dorsal ACC	± 5	10	33
A3	Subgenual ACC	± 5	34	−4

MNI, Montreal neurological institute; ACC, anterior cingulate cortex.

### Statistical analysis

All statistical analyses were performed using the Statistical Package for the Social Sciences (SPSS) software version 22.0 (IBM, Armonk, New York, NY, USA). Analyses of variance (ANOVA), two-sample *t*-tests, and chi-square tests were used to compare demographic and neurocognitive data among the three groups (HC, SCD, and aMCI groups). Making multiple comparisons increases the likelihood that a nonnegligible proportion of associations will be false positives. In order to reduce false positives, threshold-free cluster enhancement (TFCE) and family-wise error (FWE) were corrected (Smith and Nichols, 2009; Yan et al., 2016; Chen et al., 2018). It is important to note that the relevant analyses were conducted with different groups of participants.

As done in previous studies (Chen et al., 2016b), we improved statistical ability by synthesizing neuropsychological assessment data into four cognitive domains (Gu et al., 2017; Gao et al., 2018): episodic memory, information processing speed, executive function, and visuospatial function. Episodic memory data comprised Avlt-20 min DR, LMT-20 min DR, and CFT-20 min DR. Information processing speed data included DSST, TMT-A, Stroop A, and Stroop B results. Visuospatial function data were mainly extracted from CFT and CDT. Executive function data comprised VFT, DST backward, TMT-B, Stroop C, and semantic similarity. The individual raw scores for each neuropsychological test were then converted into standardized scores. The normalized Z scores were then averaged to create composite Z scores for each cognitive domain (Xie et al., 2012; Yuan et al., 2016; Chen et al., 2016b).

After controlling for age, sex, education, and GM, one-way ANOVA was used to compare FC differences among the three groups for each ACC subregion seed (1,000 permutations, *p* < 0.05, cluster size > 200 mm^3^). The two-sample *t*-test (corrected for age, sex, education, and GM) was used for *post hoc* analyses (*p* < 0.05, TFCE-FWE corrected). First, we extracted the FC of the significantly altered regions using DPABI. Correlation analysis was then used to assess the relationship between FC of the seed point changes and cognitive domains controlling for age, sex, and education level (*p* < 0.05, Bonferroni corrected).

## Results

### Participants’ demographic and neurocognitive characteristics

Table 2 provides the demographic and neurocognitive data of all participants, including 78 HC (mean age 63.56 ± 6.914), 66 SCD (mean age 65.55 ± 7.519), and 71 aMCI (mean age 65.13 ± 7.625) participants. As predicted, there were significant group differences in cognitive performance (episodic memory: *r* = 0.345, *p* < 0.001, executive function: *r* = −0.311, *p* < 0.001). The aMCI group showed decreased information processing speed and episodic memory compared with the HC and SCD groups.

**TABLE 2:** Demographics and clinical measures of healthy control (HC) and patients with subjective cognitive decline (SCD), and amnestic mild cognitive impairment (aMCI).

Characteristics	HC	SCD	aMCI	*F*-values (χ^2^)	*p*-values
			
	*n* = 78	*n* = 66	*n* = 71		
Age (years)	63.56 (6.914)	65.55 (7.519)	65.13 (7.625)	1.488	0.228
Gender (male/female)	29/49	15/51	25/46	3.899	0.142
Education level (years)	12.41 (2.637)	11.78 (2.715)	11.05 (2.938)^b^	4.51	0.012*
**Composite Z scores of each cognitive domain**					
Episodic memory	0.25 (0.90)	0.39 (0.86)	−0.63 (0.93)^b^^c^	25.943	0.000*
Information processing speed	0.28 (1.02)	0.17 (1.01)	−0.46 (0.78)^b^^c^	12.624	0.000*
Executive function	0.11 (0.15)	−0.19 (1.77)	0.06 (0.17)	1.782	0.171
Visuospatial function	−0.18 (1.58)	0.14 (0.38)	0.08 (0.27)	2.193	0.114

Data are presented as mean (standard deviation, SD). HC, healthy controls; SCD, subjective cognitive decline; aMCI, amnestic mild cognitive impairment. *Significant differences were found among HC, SCD, and aMCI subjects. Most *p*-values were obtained using ANOVA, except for gender (chi-square test). Comparisons of each paired group were conducted to further reveal the source of ANOVA difference (a: SCD vs. HC; b: aMCI vs. HC; c: aMCI vs. SCD). Bonferroni correction was applied for multiple group comparisons. This study utilized the composite Z scores to determine the level of each cognitive domain. *p < 0.05.

### Functional connectivity of anterior cingulate cortex subregions in patients with subjective cognitive decline and amnestic mild cognitive impairment

**FIGURE 2:**
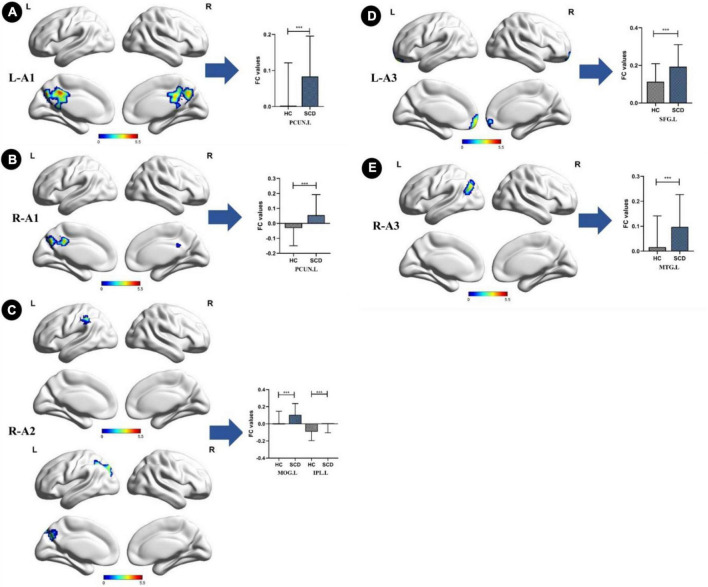
Anterior cingulate cortex subregions functional connectivity in patients with subjective cognitive decline (SCD) compared to healthy control (HC). **(A)** Functional connectivity (FC) of left caudal anterior cingulate cortex subregion (L-A1) between HC and SCD patients. A bar chart indicating the quantitative comparison of FC between these regions. **(B)** FC of right caudal anterior cingulate cortex subregion (R-A1) between HC and SCD patients. A bar chart indicating the quantitative comparison of FC between these regions. **(C)** FC of right dorsal anterior cingulate cortex subregion (R-A2) between HC and SCD patients. A bar chart indicating the quantitative comparison of FC between these regions. **(D)** FC of left subgenual anterior cingulate cortex subregion (L-A3) between HC and SCD patients. A bar chart indicating the quantitative comparison of FC between these regions. **(E)** FC of right subgenual anterior cingulate cortex subregion (R-A3) between HC and SCD patients. A bar chart indicating the quantitative comparison of FC between these regions. only the subnetworks that show between-group differences are shown here. All results are displayed after adjusting for age, sex, and education. A threshold of *p* < 0.05 was applied, with a TFCE-FWE correction with cluster size > 200 mm^3^. FC, functional connectivity; HC, healthy controls; SCD, subjective cognitive decline; PCUN.L, left precuneus; MOG.L, left middle occipital gyrus; IPL.L, left inferior parietal lobule; SFG.L, left superior frontal gyrus; MTG.L, left middle temporal gyrus. ***p < 0.05.

**FIGURE 3:**
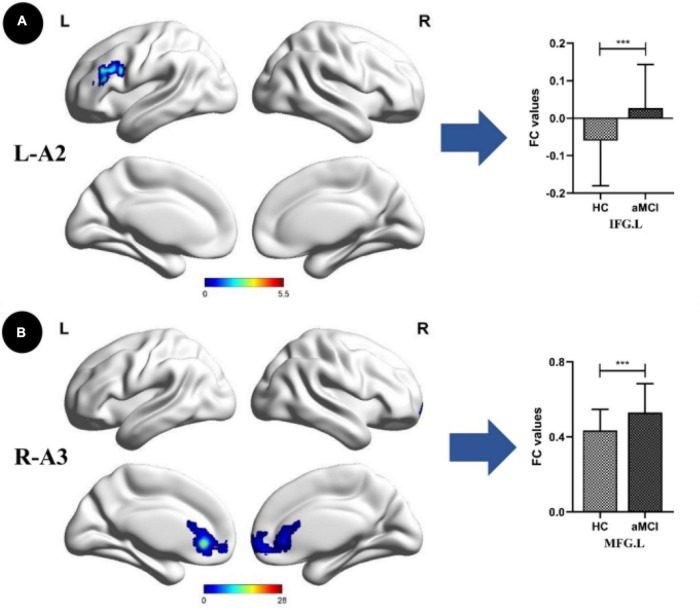
Anterior cingulate cortex subregions functional connectivity in patients with amnestic mild cognitive impairment (aMCI) compared to healthy control (HC). **(A)** Functional connectivity (FC) of left dorsal anterior cingulate cortex subregion (L-A2) between HC and aMCI patients. A bar chart indicating the quantitative comparison of FC between these regions. **(B)** FC of right subgenual anterior cingulate cortex subregion (R-A3) between HC and aMCI patients. A bar chart indicating the quantitative comparison of FC between these regions. Only the subnetworks that show between-group differences are shown here. All results are displayed alter adjusting for age, sex and education. A threshold of *p* < 0.05 was applied, with a TFCE-FWE correction with cluster size > 200 mm^3^. FC, functional connectivity; HC, healthy controls; aMCI, amnestic mild cognitive impairment; IFG.L, left inferior frontal gyrus; MFG.L, left middle frontal gyrus. ***p < 0.05.

**FIGURE 4:**
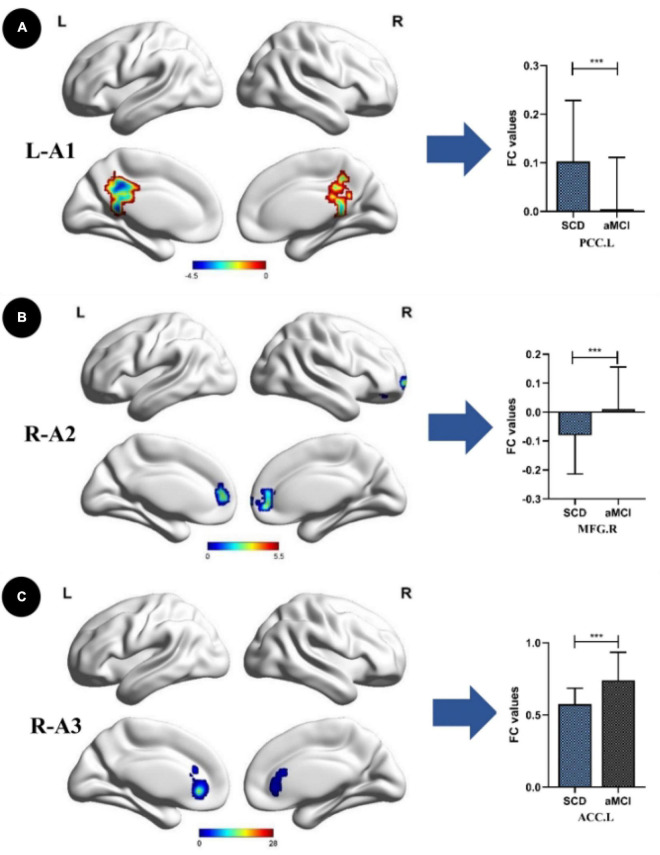
Anterior cingulate cortex subregions functional connectivity in patients with amnestic mild cognitive impairment (aMCI) compared to subjective cognitive decline (SCD). **(A)** Functional connectivity (FC) of left caudal anterior cingulate cortex subregion (L-A1) between SCD and aMCl patients. A bar chart indicating the quantitative comparison of FC between these regions. **(B)** FC of right dorsal anterior cingulate cortex subregion (R-A2) between SCD and aMCI patients. A bar chart indicating the quantitative comparison of FC between these regions. **(C)** FC of right subgenual anterior cingulate cortex subregion (R-A3) between SCD and aMCI patients. A bar chart indicating the quantitative comparison of FC between these regions. Only the subnetworks that show between-group differences are shown here. All results are displayed after adjusting for age, sex, and education. A threshold of *p* < 0.05 was applied, with a TFCE-FWE correction with cluster size > 200 mm^3^. FC, functional connectivity; SCD, subjective cognitive decline; aMCI, amnestic mild cognitive impairment; PCC.L, left posterior cingulate; MFG.R, right middle frontal gyrus; ACC.L, left anterior cingulate. ***p < 0.05.

Compared with the HC group, the SCD group showed the following changes: increased FC between the L-A1 and left PCUN, R-A1 and left PCUN, R-A2 and left MOG and left IPL, L-A3 and left SFG, and R-A3 and left MTG (*p* < 0.05, TFCE-FWE corrected, cluster size > 66 mm^3^) (Figure 2 and Table 3). Compared with the HC group, the aMCI group showed the following changes: increased FC between the L-A2 and left IFG, R-A3 and left MFG (*p* < 0.05, TFCE-FWE corrected, cluster size > 66 mm^3^; Figure 3 and Table 3). Compared with the SCD group, the FC changes in the aMCI group were as follows: increased FC between the R-A2 and right MFG as well as the R-A3 and left ACC, and decreased FC between the L-A1 and left PCC (*P* < 0.05, TFCE-FWE corrected, cluster size > 66 mm^3^; Figure 4 and Table 3).

**TABLE 3:** Comparisons of functional connectivity of anterior cingulate cortex subregions.

Brain regions	L/R	MNI			*F/T*-values	Cluster size (mm^3^)
				
		X	Y	Z		
**L-caudal ACC functional connectivity**						
**HC vs. SCD**						
Precuneus	L	−6	−42	39	5.1936	811
**SCD vs. aMCI**						
Posterior cingulate	L	−3	−39	39	−4.263	369
**R-caudal ACC functional connectivity**						
**HC vs. SCD**						
Precuneus	L	−9	−66	36	4.3885	147
**L-dorsal ACC functional connectivity**						
**HC vs. aMCI**						
Inferior frontal gyrus	L	−36	33	15	3.4944	136
**R-dorsal ACC functional connectivity**						
HC vs. SCD						
Middle occipital gyrus	L	−15	−66	36	4.5533	407
Inferior parietal lobule	L	−51	−42	48	3.7425	150
**SCD vs. aMCI**						
Middle frontal gyrus R	−15	48	6	3.853	242	
**L-subgenual ACC functional connectivity**						
HC vs. SCD						
Superior frontal gyrus	L	−9	57	−24	4.2268	282
**R-subgenual ACC functional connectivity**						
HC vs. SCD						
Middle temporal gyrus	L	−45	−75	30	4.1189	167
**HC vs. aMCI**						
Medial frontal gyrus	L	−6	33	−3	27.317	355
**SCD vs. aMCI**						
Anterior cingulate	L	−6	33	−3	25.197	121

HC, healthy controls; SCD, subjective cognitive decline; aMCI, amnestic mild cognitive impairment; L, left hemisphere; R, right hemisphere; MNI, Montreal neurological institute. The x, y, z coordinates are the primary peak locations in the MNI space. All results are displayed after adjusting for age, sex, and education at a threshold of *p* < 0.05, after applying TFCE-FEW correction with cluster size > 200 mm^3^.

### Behavioral significance of disrupted anterior cingulate cortex subregion functional connectivity

**FIGURE 5:**
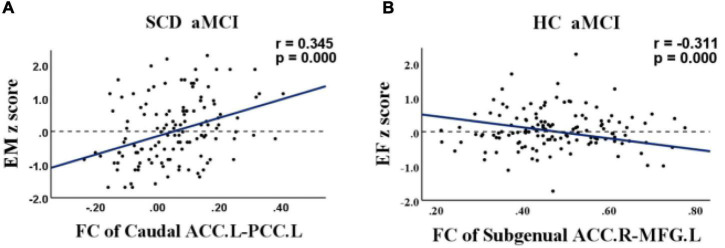
Relationship between abnormal anterior cingulate cortex subregions functional connectivity and cognition in healthy control (HC), subjective cognitive decline (SCD), and amnestic mild cognitive impairment (aMCI). **(A)** Relationship between functional connectivity (FC) of the left caudal anterior cingulate cortex and the left posterior cingulate and episodic memory in patients with SCD and aMCI. **(B)** Relationship between FC of the right subgenual anterior cingulate cortex and the left medial frontal gyrus and executive function in patients with HC and aMCI. FC, functional connectivity; HC, healthy controls; SCD, subjective cognitive decline; aMCI, amnestic mild cognitive impairment; ACC, anterior cingulate cortex; PCC, posterior cingulate; MFG, medial frontal gyrus; EM, episodic memory; EF, executive function.

For the SCD and aMCI groups, altered FC between the left caudal ACC and left posterior cingulate was positively correlated with episodic memory (*r* = 0.345, *p* < 0.001). For the HC and aMCI groups, altered FC between the right subgenual ACC and left MFG was negatively correlated with executive function (*r* = −0.311, *p* < 0.001). Age, gender, and years of education were included as covariates in these analyses (Figure 5).

## Discussion

This study aimed to explore changes in FC patterns in ACC subregions among patients on the preclinical AD spectrum and to explore how altered FC affects cognitive function. To our knowledge, this is the first study to investigate FC changes in ACC subregions during the SCD and aMCI stages. Our results showed that ACC subregion FC changes were not limited to connectivity with a single brain region but were widely distributed, including the frontal lobes, temporal lobes, parietal lobes, and occipital lobes. Secondly, correlation analysis showed that FC changes in the PCC were positively correlated with memory performance and the MFG were negatively correlated with executive function.

### Altered functional connectivity patterns of anterior cingulate cortex subregions in patients with subjective cognitive decline

Using A1 as the seed, we demonstrated increased FC with the PCUN in SCD patients compared with HCs. Similarly, using A2 as the seed, FC with the IPL and MOG increased and, when using A3 as the seed, FC with the SFG and MTG increased. It should be noted that the PCUN, IPL, and MOG are all part of the default mode network (DMN). Previous studies have shown that AD patients reduced episodic memory ability is associated with structural and functional defects of the DMN (Motomura et al., 2014), which may be due to the accumulation of amyloid plaques (Mintun et al., 2006) and reduced metabolic activity (Matsuda, 2001). In contrast, the left MOG is part of the visual network, which is related to the processing of visual memory and visual-spatial function (Bays and Husain, 2008; Weiler et al., 2014). The MTG is involved in many tasks related to word understanding and semantic cognition (Chang et al., 2015; Xu et al., 2015). Interestingly, the MTG is important for understanding visual and auditory information and is thought to use contextual knowledge to retrieve the relevant semantic information needed for these tasks (Dick et al., 2014; Jackson et al., 2016). It is worth pointing out that FC of the SFG is decreased in aMCI patients (Wang et al., 2012) but increased in SCD patients, which may be the result of patients’ ability to maintain a normal life. Our results thus suggest a potential brain basis for SCD patient’ ability to maintain multiple normal cognitive domains.

### Altered anterior cingulate cortex subregion functional connectivity patterns in amnestic mild cognitive impairment patients

When compared to the HC group, aMCI patients showed increased FC of the MFG and SFG with the A2 seed site. Currently, it is widely accepted that the MFG can be divided between dorsolateral and ventromedial regions based on anatomical connectivity and functional specialization (Rosenkilde, 1979; Kringelbach and Rolls, 2004; Zald, 2007). The prefrontal cortex has key cognitive functions related to social behavior, emotion, and motivation. The dorsolateral prefrontal cortex (DLPFC) plays important roles in working memory, goal-driven attention, task switching, planning, problem solving, and novelty seeking. The ventromedial prefrontal cortex (vmPFC) is mainly responsible for inhibition, response selection, and monitoring (Jones and Graff-Radford, 2021). The different functions of these regions broadly suggest that the MFG plays a fundamental role in internally guided cognition (Putnam and Chang, 2021). It has been previously reported that the IFG is part of a language network and is mainly responsible for declarative long-term memory (Lunsford-Avery et al., 2020). Moreover, atrophy of the IFG can predict the transition from aMCI to AD (Bokde et al., 2006). Based on the above findings, it can be speculated that increased FC with the MFG and IFG may be closely related to aMCI patients’ ability to maintain a normal daily life.

### Convergent and divergent altered functional connectivity of anterior cingulate cortex subregions in subjective cognitive decline and amnestic mild cognitive impairment patients

Our comparison of FC between the aMCI and SCD groups revealed several interesting findings. Using A1 as the seed, the FC of PCC decreased, which is the only FC decrease observed in our study. Using A2 as the seed, FC of the MFG and ACC increased. In addition, with A3 as the seed, FC with the ACC increased. Increased FC with the MFG may reflect inefficient compensation before the clinical symptoms of mild cognitive impairment appear. In other words, this suggests that MCI patients may use enhanced FC to compensate for network disruption in advanced stages of the disease (Wang et al., 2012). Interestingly, compared to the SCD and HC groups, FC with the MFG was increased in the aMCI group, which may be of clinical significance for identification of aMCI patients.

A previous functional imaging study reported that the connection strength of the DLPFC and ACC was correlated with executive function in MCI patients (Wu et al., 2014), which is consistent with our findings. The FC of the A2 seed and ACC increased, and correlation analysis showed that the increased FC of the ACC was negatively correlated with executive function (*r* = −0.311, *p* < 0.001), i.e., with increasing FC in the aMCI stage, executive function is continuously impaired. Previous studies have reported executive dysfunction and frontal lobe changes (Brandt et al., 2009; Clément et al., 2013; Kirova et al., 2015; Seo et al., 2016). Executive function includes a set of top-down cognitive processes that support goal-directed behavior (Chang et al., 2010; Diamond, 2013). It may be inferred that executive function and memory interact with each other (Chang et al., 2010). Therefore, we believe that ACC FC may be a useful predictor of AD conversion.

Another noteworthy result is that FC of the PCC decreased with the A1 seed point. As is well-known, the PCC is a key region of the post DMN (pDMN), which is mainly involved in episodic memory retrieval (Yang et al., 2017; Wang et al., 2018). Thus, our results support that FC decline of the PCC is positively correlated with memory (*r* = 0.345, *p* < 0.001), i.e., with a decline in PCC FC, memory also declines. A recent meta-analysis found that low metabolism in the PCC and PCUN are the most reliable markers for early detection and tracking of MCI to AD transformation (Ma et al., 2018). Overall, it can be concluded that decreased FC of the PCC plays an important role in the progression of AD.

In summary, this study of rsFC of the ACC subregions of preclinical spectrum AD patients showed that, compared to HCs, FC of the MFG and ACC increased, and the only region with reduced FC was the PCC. Moreover, increased MFG connectivity was positively correlated with executive function and, most importantly, decreased PCC FC was positively correlated with memory. Overall, these results raise the possibility that region-specific changes in FC, using ACC subregions as seeds, could support diagnosis of SCD and aMCI and provide new insights for clinical interventions.

### Limitations

Although our results may be of clinical significance, they are also subject to some limitations. First, significant differences in age and education levels were present among the three groups and potentially affected our results. However, to avoid the influence of these factors, we included age, education level, and gender as covariates in all statistical analyses. Therefore, we believe that our results are credible. Secondly, we studied the behavioral significance of FC abnormalities in the resting state, which has some limitations. Task-based fMRI could be utilized in future studies to verify these results.

## Conclusion

Subjective cognitive decline (SCD) and aMCI, which are part of the preclinical AD spectrum, are associated with some convergent and divergent FC alterations of ACC subregions. Patient data showed that altered FC affects cognition, including episodic memory and executive function. These results provide novel insights into tailored clinical interventions across the preclinical AD spectrum.

## Data availability statement

The raw data supporting the conclusions of this article will be made available by the authors, without undue reservation.

## Author contributions

QY, XL, and CX collected and processed the data together and wrote the manuscript. WQ, SC, YS, HW, and XZ collected the data and collated. CX and JC came up with ideas and revised the manuscript. All authors contributed to the article and approved the submitted version.

## Footnotes

1
http://www.mathworks.com/products/matlab/

2
https://www.fil.ion.ucl.ac.uk/spm/
